# A systematic review of how endocrine-disrupting contaminants are sampled in environmental compartments: wildlife impacts are overshadowed by environmental surveillance

**DOI:** 10.1007/s11356-025-36211-y

**Published:** 2025-03-15

**Authors:** Angela Simms, Kylie Robert, Ricky-John Spencer, Sarah Treby, Kelly Williams-Kelly, Candice Sexton, Rebecca Korossy-Horwood, Regan Terry, Abigail Parker, James Van Dyke

**Affiliations:** 1 Centre for Freshwater Ecosystems, Department of Environment & Genetics, School of Agriculture, Biomedicine and Environment, La Trobe University, Wodonga Campus, Wodonga, 3690 Australia; 2 School of Agriculture, Biomedicine and Environment, La Trobe University, Melbourne, 3086 Australia; 3 School of Sciences, Western Sydney University, Penrith, NSW 2751 Australia; 4 Applied Chemistry and Environmental Science, RMIT University, Melbourne, Australia; 5 School of Biosciences, The University of Melbourne, Melbourne, Australia; 6 School of Biological Sciences, Monash University, Clayton, VIC 3800 Australia

**Keywords:** Pollutant, Ecotoxicology, Endocrine disruptors, EDCs

## Abstract

Endocrine-disrupting contaminants (EDCs) are frequently monitored in environments because of their biological impacts on wildlife and humans. We conducted a systematic review using Web of Science to identify global research trends for EDC environmental sampling. Specifically, we aim to better understand geographic variation in (1) the compartment that EDCs were sampled in the environment; (2) the types of EDC sampled; and (3) the taxa that are sampled. A total of 9140 papers were found, of which 2554 were included in our review. The number of studies sampling EDCs varied between continents, with majority of research occurring in Europe, Asia, and North America. Although economy and access to technology will contribute to the number of articles published, we found the current output of research showed distinct disparities in sampling methods. Across all continents, water was the most frequently sampled compartment to determine EDC concentrations (sampled in 50–75% of studies). Wildlife was sampled far less often in studies across all continents, comprising 30% of studies at most. Pharmaceuticals were the most commonly studied chemical group, and fish were the most commonly sampled taxonomic group. Although far fewer studies sampled for EDCs in wildlife compared with abiotic compartments, these studies provide valuable information on the potential consequences of environmental EDC exposure and link environmental surveillance of EDCs with lab-measured organism-level effects. Studies that sampled only the water matrix for EDCs may be doing so as a proxy despite the large knowledge gaps on how environmental EDCs affect wildlife at varying concentrations.

**Supplementary Information:**

The online version contains supplementary material available at 10.1007/s11356-025-36211-y.

## Introduction

Anthropogenic pollution is found in even the most remote parts of the world (Puasa et al. 2021). Substantial lab-based, physiological research highlights the complex ways that pollutants affect organisms, and we are only beginning to understand the vast global impact pollution has on both biodiversity and humanity (Puckowski et al. 2016; Rzymski et al. 2020). Endocrine-disrupting contaminants (EDCs) are defined by the United States Environmental Protection Agency as “exogenous agents that interfere with the synthesis, secretion, transport, binding, action, or elimination of natural hormones in the body that are responsible for the maintenance of homeostasis, reproduction, development, and/or behaviour” (Crisp et al. 1997). Endocrine-disrupting contaminants, whether natural or synthetic, can reduce reproductive success (Marlatt et al. 2022), disrupt embryonic development and survival (Guillette et al. 2000; Hamlin and Guillette 2011), and affect thyroid, nervous, and immune functions (Colborn et al. 1993; Guillette et al. 1994).

Endocrine-disrupting contaminants originate from several anthropogenic sources and can be challenging to completely remove before entering the environment (Patel et al. 2020). Major sources of EDCs include medications, pesticides, and personal care products from hospitals, households, industries, and agriculture that largely end up in the environment after being treated in wastewater facilities or as runoff (Puri et al. 2022). Wastewater effluent contains numerous EDCs (e.g., pharmaceuticals, per- and polyfluoroalkyl substances (PFAS), alkylphenols, organochlorines, halogenated aromatic hydrocarbons, brominated flame retardants, and phthalates) that can be difficult to remove completely and therefore eventually enters the environment (Hamdhani et al. 2020; Puri et al. 2022). Agricultural effluent and runoff also contribute significantly to the contamination of waterways, with chemical residues (i.e., pesticides), and excrement of livestock/poultry containing high levels of steroid hormones and pharmaceuticals (Metcalfe et al. 2022; Puri et al. 2022).

Endocrine-disrupting contaminants have been linked to changes in wildlife populations, raising concerns about how EDCs affect the organisms within the environment and its flow on effects to the broader ecosystem (Marlatt et al. 2022). Consequently, many countries have developed regulatory guidelines for known EDCs to reduce their presence in the environment and mitigate their impacts on both wildlife and potentially, humans (Rzymski et al. 2020). Thus, the presence and concentrations of environmental EDCs are monitored. Different environmental compartments, such as water, sediment, soil, and biota, can be used to evaluate EDC concentrations. While concentrations of EDCs will vary among sample types and chemicals of interest due to differences in accumulation (or bioaccumulation) rates that can be further influenced by environmental factors (i.e., temperature, water chemistry, flow/currents, etc.) (Eljarrat et al. 2004; Liu et al. 2017; Salgueiro-González et al. 2015). In samples collected from biota, EDC concentrations may be more challenging to determine due to post-accumulation catabolism, which may result in low observed concentrations but may still have caused physiological effects on the organism (Laranjeiro et al. 2018; Olsson et al. 1994). Furthermore, the way the biota sample is collected will determine the concentration of EDCs found; for example, EDCs accumulate at different rates within different tissue samples (i.e., liver, claw, kidney, etc.), blood, and urine (Beale et al. 2022). Presumably, research has focused on concentrations of EDCs in environmental media (i.e., water, sediment), rather than investigating the occurrence of endocrine disruption in wildlife from EDC exposure (Guillette 2006). Understanding the occurrence of endocrine disruption is important (Marlatt et al. 2022), although the research needed to measure it requires more time and resources (Guillette 2006). Limited research links environmental EDC concentrations in abiotic compartments like water directly with exposure effects on wild biota, where most of our current understanding is from short-term laboratory studies on select chemical exposure to an animal, while far fewer studies have investigated the effects from chronic exposure to wildlife (Fent et al. 2006).

The objective of our global systematic review was to identify the current extent of abiotic sampling of environmental EDC concentrations, compared to direct sampling of wildlife, across continents. Currently, there are no universal guidelines due to inconsistent monitoring for environmental EDCs with regulations varying from country-specific to region-specific (Puri et al. 2022). From an environmental perspective, EDCs within the environment are not always defined by country borders; combined with the variability in the scale of regulation, we explored the variation in EDC sampling among continents as environmental EDC trends. However, we acknowledge that individual countries are faced with different challenges, and may use different monitoring methods for EDCs. We compared geographic variation in (1) the environmental compartment sampled for EDCs; (2) the types of EDC sampled; and (3) the taxa or other environmental media sampled. Environmental contaminant research may have understudied direct wildlife EDC concentrations (Colborn et al. 1993; Kraak 1998; Marlatt et al. 2022; Miller et al. 2018). The hypothesis we tested is whether EDCs are more commonly sampled in abiotic media (e.g., water, sediment) than in biotic media (e.g., wildlife), despite the fact that the impact of EDCs on wildlife is a primary focus of most EDC research. From this review, we want to advocate for more sampling of biota by highlighting the direction of global EDC environmental sampling efforts.

## Methods

### Systematic literature search

To address our aims and test our hypothesis, we undertook a systematic search of published, peer-reviewed scientific literature using Web of Science (Clarivate Analytics PLC), with article years ranging from 1980 up to those published on 14 September 2020 (date of article collection), to collate articles that sampled environmental EDCs. Web of Science was chosen due to its global credibility as a citation database and was the only source we used to collate papers due to the large volume of papers retrieved from our search, and we determined the trends we find are likely to be similar regardless of additional databases. We used the “Topic”/”TS” search string within the Core Collection of Web of Science. The search terms were chosen to target research that specifically reported on EDCs sampled from nature (i.e., water, sediment, soil, plants, and animals). Because many medical studies were generated from the original search terms, we had also incorporated additional exclusion terms in our search to filter out irrelevant studies more effectively. A total of 9140 papers were recovered using the final search terms. The search string used was “TS = ((“endocrine function*” OR estrogen OR oestrogen OR Xenoestrogen* OR Xeno-estrogen* OR “endocrine disrupting chem*” OR “endocrine disrupting pollut*” OR “hormone disrupt*” OR “endocrine disrupt*”) AND (biomark* OR bioindicat* OR water* OR effluent* OR aqua* OR bioaccumulat* OR biomagnifi* OR adsorp* OR tissue OR sediment OR blood OR serum OR plasma) AND (concentrat* OR measur* OR sampl* OR level*) AND (contamin* OR pharm* OR pollut* OR sewage) NOT (transcriptom* OR inject* OR oral*))”.

Following PRISMA guidelines for systematic reviews (Page et al. 2021), two of the authors independently screened the title and abstract from every article to determine whether each was relevant to our research question, using clearly defined inclusion and exclusion criteria. Inclusion and exclusion criteria were defined by the following: (1) Must sample potential EDC from the aquatic environment, i.e., effluent (from wastewater treatment plants, effluent from farms, etc.), water, sediment, biota (plants/animals). NOT based on artificial samples or manipulation/lab studies (NOT landfill, pools, or drinking water) measurements; (2) NOT including/using samples taken from humans and/or livestock (e.g., milk, manure); (3) NOT including reviews or modelling—UNLESS they include their own primary studies or quantitatively sample from the environment; (4) Studies must provide a quantitative result from EDC sampled; and (5) Studies are written in English. Where there were discrepancies between screeners, the paper underwent another review by the first author to finalize its inclusion or exclusion in the study. We then extracted the following data from the included papers: the continent in which the study took place; sample type (animal, plant, water, sediment, soil, or other); animal taxa sampled, where relevant (mammal, fish, amphibian, reptile, bird, arthropod, mollusc, nematode), and EDC chemical group studied (grouped following Metcalfe et al. 2022; organochlorine, halogentated aromatic hydrocarbons, brominated flame retardants, per- and polyfluoroalkyl substances, alkylphenols, phthalates, bispehnols A (and analogues), pharmaceuticals (including illicit drugs and hormones), and organotins. We analyzed three contingency tables to determine whether sample type, chemical type, and biota type deviated from the frequency expected by chance among different continents. From these contingency tables, we tested for significant differences in how continents sampled for EDCs within the environment, and the types of EDCs they investigated. Contingency tables and chi-square tests were generated using Microsoft Excel (Microsoft Corporation 2018), and built following Sokal & Rohlf, (1987). Goodness of fit was determined by calculating the chi-square values for each contingency table, and determining whether the frequency distributions in the tables were different from that predicted by random chance.

## Results

**Fig. 1:**
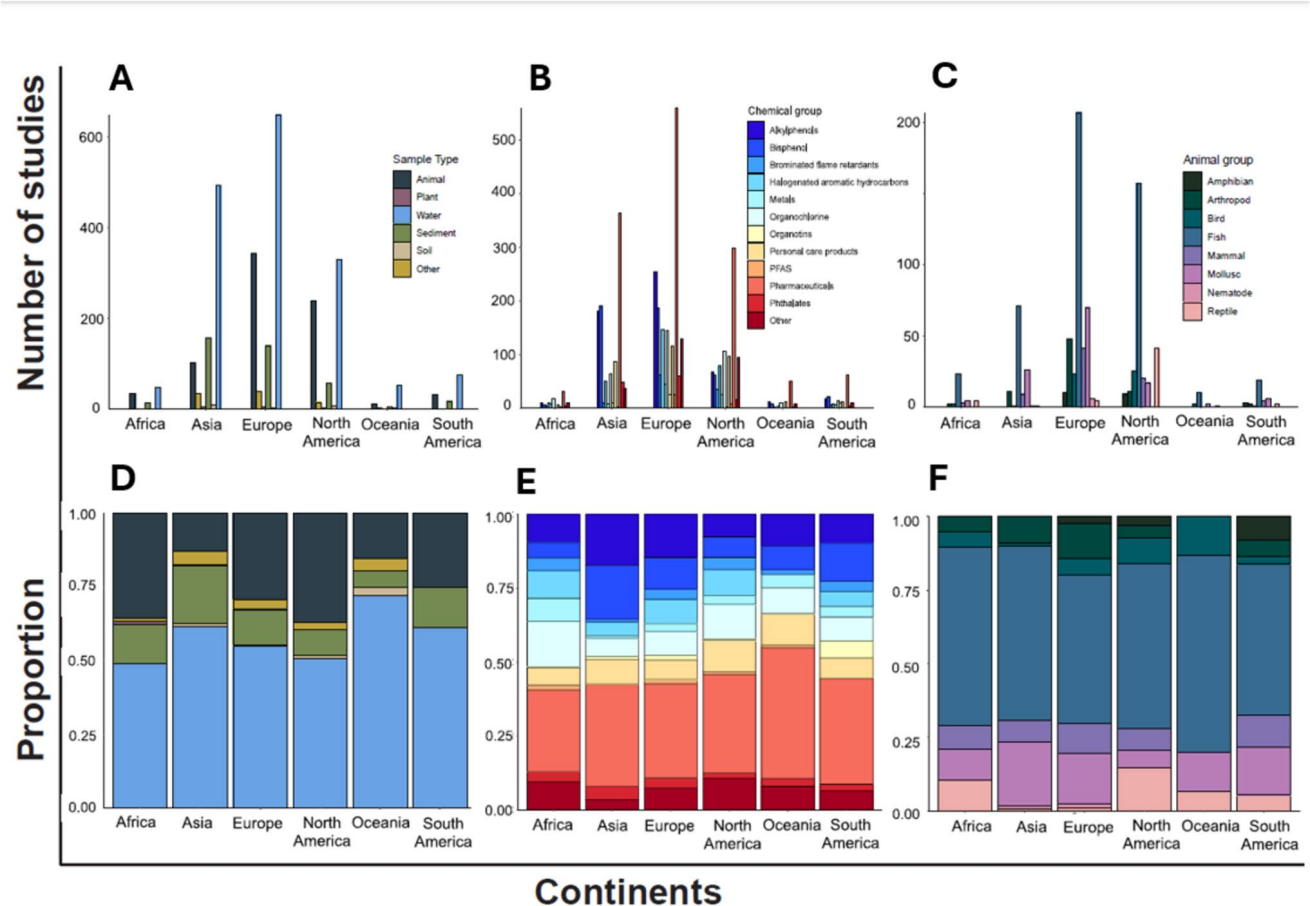
Frequency and proportion of **A**, **D** type of samples; **B**, **E** chemical group; and **C**, **F** biota type used in studies across continents to determine endocrine-disrupting contaminants in the environment

Of the 9140 papers generated, a total of 2790 were included in our review based on our specified inclusion and exclusion criteria (full list of papers provided in S1). The quantity of published literature on EDC sampling within the environment varied across continents (113–1756 studies). Antarctica had only five studies accepted in our systematic review and was subsequently combined with Oceania. Europe, Asia, and North America exhibited a significantly higher number of studies compared to Oceania, Africa, and South America. The studies conducted in different continents explored various types of environmental samples at differing rates (*X*^2^ = 170.9, d.f. = 25, *p* = < 0.0001; Fig. 1). The distribution of sample types employed in studies differed among continents (Fig. 1). Water was the most frequently studied compartment across all continents in both absolute number (48–649 studies; Fig. 1) and in proportion (50–75%; Fig. 1). Continents then sampled animals as the next most common sample compartment in studies, with the exception of Asia, which had sediment as the next most commonly sampled compartment. Continents varied in the rate they sampled animals in EDC research (11–344 studies). Animals were sampled more frequently, in approximately one-third of all studies, in Africa, Europe, and North America (Fig. 1), compared to less than a quarter of studies in South America, Oceania, and Asia. Plant (11 studies overall) and soil samples (20 studies overall) were the least commonly sampled across all continents (Fig. 1).

**Fig. 2:**
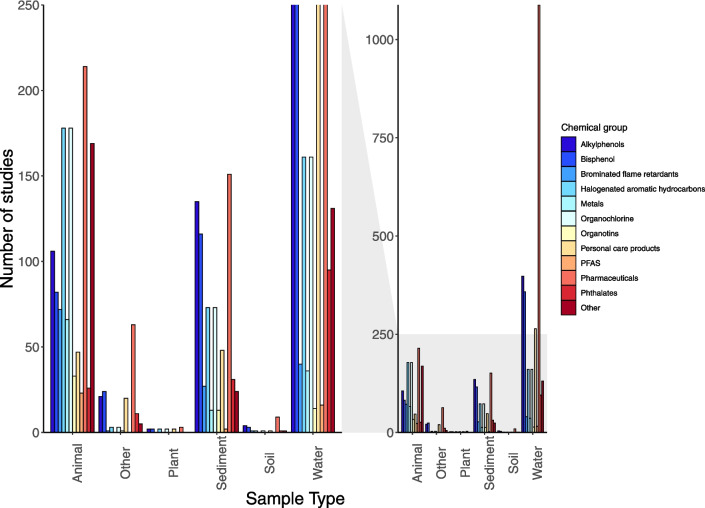
Frequency of endocrine-disrupting contaminants chemical group across sample types used in studies

Different sample types were employed to study various types of chemicals at different rates (*X*^2^ = 882.9, d.f. 55, *p* = < 0.001; Fig. 2). The majority of studies focused on sampling pharmaceuticals in water (1088 studies; Fig. 2). Following pharmaceuticals, alkylphenols, and bisphenols were the next most commonly studied chemical group, and were sampled in water (398 and 358 studies respectively; Fig. 2). In studies involving animals, the most commonly studied chemical group was pharmaceuticals (214 studies), followed by halogenated aromatic hydrocarbons (181 studies) and organochlorines (178 studies; Fig. 2).

Studies from different continents sampled different chemical groups at different rates (*X*^2^ = 335.5, d.f. = 66, *p* = < 0.001; Fig. 1). Pharmaceuticals were the most commonly studied chemical group across all continents (27–44% of studies; Fig. 1). After pharmaceuticals, the types of chemical group studied differed across continents. After pharmaceuticals, the next most studied chemical group in Africa and North America was organochlorines; in Asia, Europe, and South America, the next-most sampled were bisphenols and alkylphenols; and in Oceania, the next-most sampled were organotins followed closely by alkylphenols (Fig. 1).

The selection of animal taxa for sampling EDCs varied among continents (*X*^2^ = 127.7, d.f. = 35, *p* = < 0.0001; Fig. 1). Fish were the most commonly sampled taxonomic group across all continents (50–67% of studies; Fig. 1). Molluscs were the next most sampled group (6–21% of studies), except in North America where the next-most sampled were reptiles (15% of studies). Among the continents, only Europe included all the animal taxonomic groups in their studies (Fig. 1). Oceania exhibited the lowest diversity in animal taxa used for sampling EDCs in the environment, only sampling 4 of the 8 taxa groups (Fig. 1).

## Discussion

In our systematic review, differences were observed among studies from different continents in their approach to studying EDCs in the environment. While some differences could be attributed to the total number of articles published by researchers from each continent, we acknowledge this may be driven by a country’s economy, accessibility to funding, and technology. Overall, we still find that the current output of research showed distinct disparities in sampling methods and focus on specific EDCs. Despite these differences, certain trends were consistent across all continents. For instance, water was the most commonly sampled compartment, and the majority of studies examined the concentrations of pharmaceuticals in their samples.

Notably, continents differed in their frequencies of studying EDC samples from biota, although there was a common emphasis on fish as the primary animal group of interest in EDC research, likely due to being a global food source. It is worth mentioning that numerous laboratory studies were excluded from our systematic review due to their lack of environmental sampling or replication. Although laboratory studies can provide an initial insight into EDC dose responses in different animals, they may fail to replicate the level of exposure found in the wild, including the complex mixture of EDCs and the specific types of exposure (e.g., concentration and duration) encountered by wildlife (Miller et al. 2018). Additionally, it is important to note that EDCs are known to exhibit nonmonotonic dose responses, where greater effects can be observed at low doses that may not be apparent at higher doses (Vandenberg et al. 2012). While we recognize the importance of lab studies increasing our understanding of the potential impacts on wildlife to particular concentrations of EDCs, the complexities of environmental EDC exposure are difficult to replicate.

### Differences in EDCs and sample types studied

Our systematic review shows that wildlife was sampled far less commonly than water in EDC research. Water was consistently the dominant sample type used across all continents, but different continents did not study EDCs in water at the same rates. Three-quarters of studies from Oceania sampled water, which was proportionally higher than other continents. The water matrix within an aquatic environment is considered to be the main source of EDC exposure to wildlife and humans (Gonsioroski et al. 2020); therefore, it is not surprising that water is most commonly sampled to monitor environmental EDC concentrations. Studies measuring EDCs in animals also varied in their proportion between continents. North America and Africa had the highest proportions of studies examining EDCs in animals (~ 33%). Sampling animals provides some clarity on how environmental EDC exposure may be impacting wildlife (Kloas 2002; Marlatt et al. 2022; Tyler et al. 1998). As animals can metabolize some EDCs, concentrations found within animals may not necessarily reflect the concentration that the animal has been exposed to, and instead animals may experience physiological and behavioral changes that cannot be easily attributed to EDC causes.

Sediment and plants were sampled the least in EDC research. Given the ease in which sediment can be collected, sample rates were surprisingly low, but variable, across continents. Plants were sampled in only 11 studies across all continents, where the low sampling efforts may be due to their ability to accumulate EDCs but at different rates depending on the species. For example, the invasive curly leaf pond weed (*Potamogeton illinoensis*) has demonstrated greater accumulation of estrogenic compounds (averaging 66%) and bisphenol A (94%) than a native pondweed (Trueman and Erber 2013).

### Continental differences in EDCs studied

Since many pharmaceuticals are designed to be endocrine disruptors (i.e., oral contraceptives and hormone replacement therapy), they were, unsurprisingly, the most frequently studied chemical group across all continents. After pharmaceuticals, the next most commonly studied EDC differed among continents. In Asia, research on EDCs focused especially on bisphenol and alkylphenols. Bisphenol A (BPA) use for manufacturing plastics has grown substantially in Asia, particularly in China (Huang et al. 2012). Meanwhile, alkylphenols are still used widely in some parts of Asia for manufacturing, despite their ban in many countries (Bergé et al. 2012; Duan et al. 2014). The high use of BPA, and continued use of alkylphenols, likely explains why they are a commonly studied EDCs in Asia. For North America, it is surprising how few studies examined BPA since the continent produces approximately 22.9% of the world’s total (Huang et al. 2012). For alkylphenols, it is similarly unusual that we found North America to have far fewer studies on the chemical group; as although regulations have aimed to decrease concentrations within the environment, the levels being recovered are still of environmental significance (Bergé et al. 2012). The next most commonly studied chemical group in North America was organochlorine, followed closely by personal care products (PCPs). Organochlorine research has shown some of the best evidence for its endocrine-disrupting potential on organisms, where exposure in the environment has been extensively studied in reptiles (particularly alligators) in North America (Guillette et al. 2000; Marlatt et al. 2022). Organochlorides were also the second most commonly studied chemical group in Africa, and had a higher proportion of research undertaken compared to other continents. Organochlorines are often found in pesticides, and in African nations in particular, there is a lack of pesticide regulation and documented unsustainable farming practices that have led to concerns for community and environmental exposure (Williamson et al. 2008). The second most commonly studied chemical groups for Oceania was PCPs, and for South America was BPA; however, the reasoning for why they have been so heavily studied in these particular continents is unclear. It may be because there are far fewer studies coming from Oceania and South America, thus a lack of meaningful trends for these continents, with fewer than 100 studies looking at pharmaceuticals and < 50 studies for any other chemical group. Therefore, the proportion of chemical groups studied beyond pharmaceuticals may not necessarily be most important to those continents.

### Differences in wildlife taxa studied

Environmental EDCs have profound impacts on exposed organisms when present at sufficient concentrations (Marlatt et al. 2022). Fish were the most studied taxonomic group in EDC research across all continents. Aquatic biota, including fish, may be more vulnerable to EDC impacts due to continuous exposure in contaminated waterways, and fish have provided the strongest evidence that EDCs are impacting wild populations (Aris et al. 2014). Some of the earliest substantial evidence of wildlife impacts from EDC exposure was in the 1990s in flounder (*Platichthys flesus*) downstream of a sewage treatment facility in the UK (Allen et al. 1999). Male flounder were found to have significantly elevated vitellogenin concentrations and reproductive abnormalities when sampled from waterways exposed to sewage effluent (Allen et al. 1999). Fish are an important food source for many communities globally. Therefore, understanding how EDCs are impacting fish stocks, and the potential bioaccumulation of EDCs within fish, which may biomagnify in humans consuming fish, is crucial for both community health and food security (Aris et al. 2014; Lv et al. 2019). These studies demonstrate the importance of linking environmental surveillance of EDCs with lab-measured organism-level effects, rather than leaving environmental data to stand on their own as proxies of impacts.

Other aquatic organisms are likely to be similarly impacted by EDCs but studied far less often. Amphibians were one of the least-studied vertebrates for EDC concentrations, although existing research suggests they are just as vulnerable as fish to EDC exposure, and they are declining globally (Kloas 2002). Amphibians are classic model organisms in endocrinology and developmental biology research, providing a solid foundation for mechanistic EDC research in other wild animals (Kloas 2002). Hayes et al. (2002) demonstrated the feminization of leopard frogs (*Rana pipiens*) associated with waterborne atrazine (a common herbicide) in parts of the USA. Their study also found a higher rate of male leopard frogs having reproductive abnormalities in areas where atrazine sales were greater than 0.4 kg/km^−2^ and present at 0.2 ppb within waterbodies (Hayes et al. 2002). Other studies have provided further supportive evidence that EDCs impact frog populations. For example, the proportion of intersex cricket frogs (*Acris crepitans*) in the USA increased during the industrial growth era with the extensive use of EDCs like PCBs, DDT, and organochlorine pesticides (Reeder et al. 2005). Even in suburban environments, where sources of EDCs are less clear, sensitive frog populations still became feminized from the exposure (Lambert et al. 2015).

Reptiles were understudied globally, with almost 80% of reptile EDC studies come from North America. In North America, the physiological impacts of EDCs on aquatic reptiles like chelonians and crocodilians have been documented (Guillette 2006; Irwin et al. 2001). Many reptile species have high site fidelity, carnivorous diets, and long life spans that presumably heighten their EDC exposure, and consequently, are excellent sentinel species for examining long-term EDC exposure (Hopkins 2000). The most extensively studied reptile are the alligators in Lake Apopka (FL, USA), a pesticide-contaminated lake, where their exposure to EDCs has led to altered plasma hormone concentrations and developmental defects that are shown in hatchlings (Guillette et al. 2000).

To date, literature on EDC bioaccumulation and biomagnification in animals is limited to select species and chemicals, with evidence to support its prevalence within wildlife; however, knowledge is restricted with how it manifests differently across species. From the limited literature available, bioaccumulation of EDCs has been documented in fish (Wang and Gardinali 2013), crustaceans (Vernouillet et al. 2010), algae (Vernouillet et al. 2010), turtles (Beale et al. 2022), and molluscs (de Solla et al. 2016). Understandably, from a human-centered perspective, there is greater importance in understanding bioaccumulation in seafood to ensure human populations are not consuming potentially high doses of EDCs (Ebele et al. 2017). Physiological differences across species will likely underlie variation in how contaminants bioaccumulate and biomagnify (Lv et al. 2019; Puckowski et al. 2016). For example, invertebrates are presumed to have greater rates of EDC bioaccumulation than vertebrates since they have a greater surface-to-volume ratio (Cuvillier-hot and Lenoir 2020). Invertebrates are generally easier to study than vertebrates, since they are smaller and more abundant, and usually have fewer regulatory permit requirements. However, there are limitations to our understanding of EDC impacts on invertebrates, due to our current inadequate understanding of their endocrinology, which is fundamental for EDC research (Ford and Leblanc 2020a; Hutchinson 2007). For abiotic compartments to be an effective proxy to understand when concentrations of EDCs are excessive or likely to cause biological harm, more research is needed investigating the impacts of environmental EDC exposure across a range of wildlife.

### Conclusion

Our systematic review highlights the extent of environmental EDC research to date, where less than a third of studies have examined EDC exposure on wildlife, or have sampled biota within the environment. Some of the earliest studies on the impacts of EDC exposure on wildlife occurred in the 1970s (Blaber 1970; Marlatt et al. 2022). Since then, studies have prioritized monitoring water to determine EDC concentrations within the environment as a proxy for wildlife exposure, and far fewer studies have directly measured EDC accumulation or concentrations within wildlife. Access to funding and technology will be a limiting factor for developing nations; however, we find that developed nations (i.e., Oceania, which includes Australia and New Zealand) are very similar to continents that likely face more resource-restricted limitations. Some of the limited progress on EDC exposure and possible reluctance to sample biota may be explained by the knowledge gaps on foundational endocrinology for some organisms (Ford and Leblanc 2020b), and the lack of standardization of analytical methods in ecotoxicology (Miller et al. 2018).

A critical area for future research is to develop baselines for converting abiotic EDC concentrations into dose–response curves for key organisms of local interest, which could then act as indicator or sentinel species for a given environment. Such baselines would provide two clear improvements over current sampling, which appears to be relatively unstrategic, at least on a global scale. First, having a baseline to refer to would greatly improve the relevancy of abiotic-only proxy sampling, especially in regions or systems where examining EDC exposure within wildlife more directly is prohibitively expensive. Second, baselines would allow calibration of the EDC concentrations used in laboratory studies aiming to investigate real-world implications of EDC exposure in wildlife, to prevent both over- and under-estimation of potential EDC concentrations and ultimately impacts.

## Supplementary Information

Below is the link to the electronic supplementary material.

Supplementary file1 (XLSX 2047 KB)

## Data Availability

The list of accepted articles used for data analysis is provided in the Supplementary Material of the manuscript. No data has been deposited in online databases. All data supporting the findings of this study are available within the paper and its Supplementary Information. A full list of accepted reviewed papers is provided in Supplementary S1.

## References

[CR1] Allen Y, Matthiessen P, Scott A (1999) The extent of oestrogenic contamination in the UK estuarine and marine environments – further surveys of flounder. Sci Total Environ 233(1-3):5–20 10.1016/s0048-9697(99)00175-8 10492895

[CR2] Aris AZ, Shamsuddin AS, Praveena SM (2014) Occurrence of 17-ethynylestradiol (EE2) in the environment and effect on exposed biota: a review. Environ Int 69:104–119 10.1016/j.envint.2014.04.011 24825791

[CR3] Beale DJ, Hillyer K, Nilsson S, Limpus D, Bose U, Broadbent JA, Vardy S (2022) Science of the total environment bioaccumulation and metabolic response of PFAS mixtures in wild-caught freshwater turtles (Emydura macquarii macquarii) using omics-based ecosurveillance techniques. Sci Total Environ 806:151264. 10.1016/j.scitotenv.2021.151264 34715216 10.1016/j.scitotenv.2021.151264

[CR4] Bergé A, Cladière M, Gasperi J, Coursimault A, Tassin B, Moilleron R (2012) Meta-analysis of environmental contamination by alkylphenols. Environ Sci Pollut Res 19(9):3798–3819. 10.1007/s11356-012-1094-7 10.1007/s11356-012-1094-7 22864754

[CR5] Blaber SJM (1970) The occurrence of a penis-like outgrowth behind the right tentacle in spent females of Nucella lapillus. J Molluscan Stud 39:231–233

[CR6] Colborn T, Vom Saal FS, Soto AM (1993) Developmental effects of endocrine-disrupting chemicals in wildlife and humans. Environ Health Perspect 101(5):378–384. 10.1289/ehp.93101378 8080506 10.1289/ehp.93101378 PMC1519860

[CR7] Crisp TM, Clegg ED, Cooper RL (1997) Special report on environmental endocrine disruption: an effects assessment and analysis. Risk Assessment Forum. U.S. Environmental Protection Agency. Washington, D.C, 21(3):12–42. http://downloads.esri.com/archydro/archydro/Doc/Overview of Arc Hydro terrain preprocessing workflows.pdf%0A10.1016/j.jhydrol.2017.11.003%0Ahttp://sites.tufts.edu/gis/files/2013/11/Watershed-and-Drainage-Delineation-by-Pour-Point.pdf%0Awww

[CR8] Cuvillier-hot V, Lenoir A (2020) Molecular and cellular endocrinology invertebrates facing environmental contamination by endocrine disruptors : novel evidences and recent insights. Mol Cell Endocrinol 504(January 2019):110712. 10.1016/j.mce.2020.110712 31962147 10.1016/j.mce.2020.110712

[CR9] de Solla SR, Gilroy EAM, Klinck JS, King LE, McInnis R, Struger J, Backus SM, Gillis PL (2016) Bioaccumulation of pharmaceuticals and personal care products in the unionid mussel Lasmigona costata in a river receiving wastewater effluent. Chemosphere 146:486–496. 10.1016/j.chemosphere.2015.12.022 26741555 10.1016/j.chemosphere.2015.12.022

[CR10] Duan X, Li Y, Li X, Zhang D, Gao Y (2014) Chemosphere alkylphenols in surface sediments of the Yellow Sea and East China Sea inner shelf : occurrence, distribution and fate. Chemosphere 107:265–273. 10.1016/j.chemosphere.2013.12.054 24411839 10.1016/j.chemosphere.2013.12.054

[CR11] Ebele AJ, Abdallah MA, Harrad S (2017) Pharmaceuticals and personal care products (PPCPs) in the freshwater aquatic environment. Emerg Contam 3(1):1–16. 10.1016/j.emcon.2016.12.004

[CR12] Eljarrat M, Alda M, Barceló D (2004) Endocrine disrupting compounds and other emerging contaminants in the environment: a survey on new monitoring strategies and occurrence data. Anal Bioanal Chem 374:549–562 10.1007/s00216-003-2184-7 12955281

[CR13] Fent K, Weston AA, Caminada D (2006) Ecotoxicology of human pharmaceuticals, 76:122–159.10.1016/j.aquatox.2005.09.009 10.1016/j.aquatox.2005.09.009 16257063

[CR14] Ford AT, Leblanc GA (2020a) Endocrine disruption in invertebrates: a survey of research progress. Environ Sci Technol, 54. 10.1021/acs.est.0c04226 10.1021/acs.est.0c04226 33050691

[CR15] Ford AT, Leblanc GA (2020b) Endocrine disruption in invertebrates: a survey of research progress.10.1021/acs.est.0c04226 10.1021/acs.est.0c04226 33050691

[CR16] Gonsioroski A, Mourikes VE, Flaws JA (2020) Endocrine disruptors in water and their effects on the reproductive system. Int J Mol Sci 21:1929 32178293 10.3390/ijms21061929 PMC7139484

[CR17] Guillette LJ (2006) Monograph endocrine disrupting contaminants — beyond the dogma. Environ Health Perspect 114(January 2005):9–12. 10.1289/ehp.8045 16818240 10.1289/ehp.8045 PMC1874172

[CR18] Guillette LJ, Gross TS, Masson GR, Matter JM, Percival HF, Woodward AR (1994) Developmental abnormalities of the gonad and abnormal sex-hormone concentrations in juvenile alligators from contaminated and control lakes in Florida. Environ Health Perspect 102(8):680–688. 10.2307/3432198 7895709 10.1289/ehp.94102680 PMC1567320

[CR19] Guillette L, Crain DA, Gunderson MP, Kools SAE, Milnes MR, Orlando EF, Rooney AA, Woodward AR (2000) Alligators and endocrine disrupting contaminants. Am Zool 452:438–452

[CR20] Hamdhani H, Eppehimer DE, Bogan MT (2020) Release of treated effluent into streams : a global review of ecological impacts with a consideration of its potential use for environmental flows. Freshw Biol 65:1657–1670. 10.1111/fwb.13519

[CR21] Hamlin HJ, Guillette LJ Jr (2011) Embryos as targets of endocrine disrupting contaminants in wildlife. Birth Defects Research 93(1):19–33. 10.1002/bdrc.20202 21425439 10.1002/bdrc.20202

[CR22] Hayes T, Haston K, Tsui M, Hoang A, Haeffele C (2002) Feminization of male frogs in the wild. Nature 419:895–896 12410298 10.1038/419895a

[CR23] Hopkins WA (2000) Reptiles toxicology: challenges and opportunities on the last frontier in vertebrate ecotoxicology. Environ Toxicol 19(10):2391–2393

[CR24] Huang YQ, Wong CKC, Zheng JS, Bouwman H, Barra R, Wahlstrom B, Neretin L, Wong MH (2012) Bisphenol A (BPA) in China: a review of sources, environmental levels, and potential human health impacts. Environ Int 42(SI):91–99. 10.1016/j.envint.2011.04.010 21596439 10.1016/j.envint.2011.04.010

[CR25] Hutchinson TH (2007) Small is useful in endocrine disrupter assessment — four key recommendations for aquatic invertebrate research. Ecotoxicology 16:231–238. 10.1007/s10646-006-0107-z 17219089 10.1007/s10646-006-0107-z

[CR26] Irwin LK, Gray S, Oberdo E (2001) Vitellogenin induction in painted turtle, Chrysemys picta, as a biomarker of exposure to environmental levels of estradiol. Aquat Toxicol 55:49–60 11551621 10.1016/s0166-445x(01)00159-x

[CR27] Kloas W (2002) Amphibians as a model for the study of endocrine disruptors. Int Rev Cytol 216:1–57. 10.1016/S0074-7696(02)16002-5 12049206 10.1016/s0074-7696(02)16002-5

[CR28] Lambert MR, Giller GSJ, Barber LB, Fitzgerald KC, Skelly DK (2015) Suburbanization, estrogen contamination, and sex ratio in wild amphibian populations. Proc Natl Acad Sci USA 112(38):11881–11886. 10.1073/pnas.1501065112 26372955 10.1073/pnas.1501065112 PMC4586825

[CR29] Laranjeiro F, Sánchez-Marín P, Oliveira IB, Galante-Oliveira S, Barroso C (2018) Fifteen years of imposex and tributyltin pollution monitoring along the Portuguese coast. Environ Pollut 232:411–421. 10.1016/j.envpol.2017.09.056 28986082 10.1016/j.envpol.2017.09.056

[CR30] Liu D, Wu S, Xu H, Zhang Q, Zhang S, Shi L, Yao C, Liu Y, Cheng J (2017) Distribution and bioaccumulation of endocrine disrupting chemicals in water, sediment and fishes in a shallow Chinese freshwater lake: implications for ecological and human health risks. Ecotoxicol Environ Saf 140(March):222–229. 10.1016/j.ecoenv.2017.02.045 28267651 10.1016/j.ecoenv.2017.02.045

[CR31] Lv Y, Yao L, Wang L, Liu W, Zhao J (2019) Chemosphere bioaccumulation, metabolism, and risk assessment of phenolic endocrine disrupting chemicals in specific tissues of wild fish. Chemosphere 226:607–615. 10.1016/j.chemosphere.2019.03.187 30954895 10.1016/j.chemosphere.2019.03.187

[CR32] Marlatt VL, Bayen S, Delb G, Martyniuk J, Metcalfe CD, Parent L, Rwigemera A, Thomson P, Van Der Kraak G, Armand C, Sant F (2022) Impacts of endocrine disrupting chemicals on reproduction in wildlife and humans. Environ Res 208:112584. 10.1016/j.envres.2021.112584 34951986 10.1016/j.envres.2021.112584

[CR33] Metcalfe CD, Bayen S, Desrosiers M, Muñoz G, Sauvé S, Yargeau V (2022) An introduction to the sources, fate, occurrence and effects of endocrine disrupting chemicals released into the environment. Environ Res 207:112658. 10.1016/j.envres.2021.112658 34990614 10.1016/j.envres.2021.112658

[CR34] Miller TH, Bury NR, Owen SF, Macrae JI, Barron LP (2018) A review of the pharmaceutical exposome in aquatic fauna. Environ Pollut 239:129–146. 10.1016/j.envpol.2018.04.012 29653304 10.1016/j.envpol.2018.04.012 PMC5981000

[CR35] Olsson M, Karlsson B, Ahnland E (1994) Diseases and environmental contaminants in seals from the Baltic and the Swedish west coast. Sci Total Environ 9697(94) 10.1016/0048-9697(94)90089-2 7973608

[CR36] Page MJ, Mckenzie JE, Bossuyt PM, Boutron I, Hoffmann TC, Mulrow CD, Shamseer L, Tetzlaff JM, Akl EA, Brennan SE, Chou R, Glanville J, Grimshaw JM, Mayo-wilson E, Mcdonald S, Mcguinness LA, Stewart LA, Thomas J, Tricco AC, … Moher D (2021) The PRISMA 2020 statement : an updated guideline for reporting systematic reviews. Int J Surg 88(March). 10.1016/j.ijsu.2021.105906 10.1016/j.ijsu.2021.105906 33789826

[CR37] Patel N, Khan MZA, Shahane S, Rai D, Chauhan D, Kant C, Chaudhary VK (2020) Emerging pollutants in aquatic environment : source, effect, and challenges in biomonitoring and bioremediation- a review. Pollution 6(1):99–113. 10.22059/poll.2019.285116.646

[CR38] Puasa NA, Zulkharnain A, Verasoundarapandian G, Wong C, Nabilah K, Zahri M, Merican F, Shaharuddin NA, Gomez-fuentes C, Ahmad SA (2021) Effects of diesel, heavy metals and plastics pollution on penguins in Antarctica: a review. Animals 11. 10.3390/ani11092505 Academic 10.3390/ani11092505 PMC8465831 34573474

[CR39] Puckowski A, Mioduszewska K, Łukaszewicz P, Borecka M, Caban M, Maszkowska J, Stepnowski P (2016) Bioaccumulation and analytics of pharmaceutical residues in the environment : a review. J Pharm Biomed Anal 127:232–255. 10.1016/j.jpba.2016.02.049 26968887 10.1016/j.jpba.2016.02.049

[CR40] Puri M, Gandhi K, Kumar MS (2022) A global overview of endocrine disrupting chemicals in the environment : occurrence, effects, and treatment methods. Int J Environ Sci Technol 0123456789. 10.1007/s13762-022-04636-4

[CR41] Reeder AL, Ruiz MO, Pessier A, Brown LE, Levengood JM, Phillips CA, Wheeler MB, Warner RE, Beasley VR (2005) Intersexuality and the cricket frog decline: historic and geographic trends. Environ Health Perspect 113(3):261–265 10.1289/ehp.7276 PMC1253749 15743712

[CR42] Rzymski P, Drewek A, Klimaszyk P, Rzymski P, Drewek A, Klimaszyk P (2020) Pharmaceutical pollution of aquatic environment : an emerging and enormous challenge. Limnol Rev 17(2):97–107. 10.1515/limre-2017-0010

[CR43] Salgueiro-González N, Turnes-Carou I, Besada V, Muniategui-Lorenzo S, López-Mahía P, Prada-Rodríguez D (2015) Occurrence, distribution and bioaccumulation of endocrine disrupting compounds in water, sediment and biota samples from a European river basin. Sci Total Environ 529:121–130. 10.1016/j.scitotenv.2015.05.048 26005755 10.1016/j.scitotenv.2015.05.048

[CR44] Sokal RR, Rohlf FJ (1987) Introduction to biostatistics (second edi). Dover Publications

[CR45] Trueman RJ, Erber L (2013) Invasive species may offer advanced phytoremediation of endocrine disrupting chemicals in aquatic ecosystems. Emirates J Food Agric 25(9):648–656. 10.9755/ejfa.v25i9.16393

[CR46] Tyler CR, Jobling S, Sumpter JP (1998) Endocrine disruption in wildlife: a critical review of the evidence. Crit Rev Toxicol 28(4):319–361. 10.1080/10408449891344236 9711432 10.1080/10408449891344236

[CR47] Van Der Kraak G (1998) Observations of endocrine effects in wildlife with evidence of their causation. Pure Appl Chem 70(9):1785–1794

[CR48] Vandenberg LN, Colborn T, Hayes TB, Heindel JJ, Jacobs DR Jr, Lee D-H, Shioda T, Soto AM, vom Saal FS, Welshons WV, Zoeller RT, Myers JP (2012) Hormones and endocrine-disrupting chemicals: low-dose effects and nonmonotonic dose responses. Endocr Rev 33(3):378–455. 10.1210/er.2011-1050 22419778 10.1210/er.2011-1050 PMC3365860

[CR49] Vernouillet G, Eullaffroy P, Lajeunesse A, Blaise C, Gagné F, Juneau P (2010) Chemosphere toxic effects and bioaccumulation of carbamazepine evaluated by biomarkers measured in organisms of different trophic levels. Chemosphere 80(9):1062–1068. 10.1016/j.chemosphere.2010.05.010 20557923 10.1016/j.chemosphere.2010.05.010

[CR50] Wang J, Gardinali PR (2013) Uptake and depuration of pharmaceuticals in reclaimed water by mosquito fish (Gambusia holbrooki): a worst-case, multiple-exposure scenario. Environ Toxicol Chem 32(8):1752–1758. 10.1002/etc.2238 23595768 10.1002/etc.2238

[CR51] Williamson S, Ball A, Pretty J (2008) Trends in pesticides use and drivers for safer pest management in four African countries. Crop Prot 27:1327–1334

